# Evaluation of fatigue, load and the quality of chest compressions by bystanders in hot and humid environments

**DOI:** 10.1016/j.resplu.2024.100818

**Published:** 2024-10-30

**Authors:** Haruka Takahashi, Kensuke Suzuki, Yohei Okada, Satoshi Harada, Hiroyuki Yokota, Marcus Eng Hock Ong, Satoo Ogawa

**Affiliations:** a The Graduate School of Medical & Health Science, Nippon Sport Science University, Japan; b Department of Preventive Service, Kyoto University Graduate School of Medicine, Japan; c Health Services and System Research, Duke-NUS Medical School, National University of Singapore, Singapore

**Keywords:** Bystander CPR, Physiological load, Hot Temperature, Fatigue, Heat-related illness

## Abstract

**Background:**

This study aimed to investigate the physiological load on bystanders during cardiopulmonary resuscitation (CPR) and the quality of chest compressions in hot and humid environments.

**Methods:**

This prospective experimental study compared the physical load and quality of chest compressions among healthy volunteers who performed 10 min chest compression in a climate chamber under normal conditions (for Tokyo) (Wet Bulb Globe Temperature [WBGT] 21 °C) and hot and humid conditions (WBGT 31 °C). The primary outcome was the depth of chest compressions over a 10-minute period. Secondary outcomes included the volunteer’s heart rate (HR), core body temperature (BT), Borg scale for assessing fatigue, and blood lactate levels. Data were analyzed using two-way repeated measures analysis of variance (ANOVA) and paired t-tests.

**Results:**

Out of 31 participants, 29 participants (mean [SD] age: 21[0.7], male: 21 [70.5 %]) were included in the analysis. For WBGT 21 °C and 31 °C, the mean chest compression depth at 10 min was not statistically difference (the depth of chest compression: 52.2 mm and 51.5 mm (p = 0.52)). At 10 min, heart rate and core temperature were 126 vs. 143 bpm, and 37.4℃ vs. 37.5℃ for WBGT 21℃ vs. WBGT 31℃ (mean differences: 17 bpm [95 % CI: 7.7–26.3], 0.1℃ [95 % CI: −0.1–0.3]). At the end, Borg scale was 16 vs. 18 and lactate levels were 3.9 vs. 5.1 mmol/L (mean differences: 2 [95 % CI: 1–3], 1.2 mmol/L [95 % CI: 0.1–2.3]).

**Conclusion:**

there was no significant difference in the depth of chest compression of paramedic students under the conditions between WBGT 31℃ and WBGT 21℃. For secondary outcomes, the lactate and fatigue of bystanders increased under WBGT 31℃ compared to WBGT 21℃. Further research is needed on CPR in hot and humid environments.

## Introduction

Out-of-hospital cardiac arrest (OHCA) is a life-threatening condition with high mortality and morbidity. To save the lives of OHCA patients, it is crucial for bystanders to perform continuous high-quality cardiopulmonary resuscitation (CPR) until paramedics arrive at scene^1^, ^2^. The quality of CPR is influenced by several factors, such as the background, experience of the bystander, fatigue, or situational factors. Previous studies have reported that CPR quality decreases due to factors such as the duration of chest compressions, bystanders mask usage, gender, body weight, and fatigue^3^, ^4^, ^5^, ^6^, ^7^, ^8^, ^9^, ^10^, ^11^. Accordingly, we believe that investigation of the factors associated with bystander CPR quality and fatigue of bystander are related to the prognosis of cardiac arrest patients.

We hypothesize that a hot and humid environment is one of the factors affecting the quality of CPR and fatigue of bystander. In recent years, climate change has made heat stress a global issue. In the field of sports, it is well-documented that exercising in a hot and humid environment significantly worsens performance and increases heart rate and body temperature, increasing the physiological load on athletes. While numerous studies have focused on the sports field, there is a lack of research on how the environment affects the quality of CPR, physiological load and fatigue for bystander CPR. 93.1 % of sudden cardiac arrests related to sports activity occur in outdoor settings, and approximately 30 % of them happen during the summer.　The potential influence of hot and humid environments on the quality of bystander CPR definitely needs to be investigated^12^.

This study aimed to investigate the quality of chest compressions and the physiological load on bystanders during CPR in hot and humid environments.

## Materials and Methods

### Study design

This was a prospective experimental study comparing the quality of chest compressions and physical load among healthy volunteers performing CPR under normal conditions, with Wet Bulb Globe Temperature (WGBT) 21℃, and hot and humid conditions with WGBT 31℃^13^. We have chosen this design as we were concerned that the randomized crossover design may have the risk of bias due to carryover effect and the operational constraints of using the climate chamber. This study was conducted with the approval of the Ethics Review Committee for Research Involving Human Subjects at Nippon Sport Science University (Approval Number: 021-H065).

### Setting and participants

This study setting was a climate chamber at Nippon Sport Science University, which can control the environmental temperature and humidity. We included adult healthy volunteers from the university. The healthy volunteers were young individuals aged 20 to 22 with no physical or health issues. The sample size for this study was calculated based on the following assumption referring to the previous research by Cekmen, B. et al^14^. We assumed a mean difference of 4.9 mm standard deviation of 6.0 mm in chest compression depth between WBGT 21℃ and WBGT 31℃. Using a power of 0.8, a significance level of 0.05, and a two-sided test, the required sample size was determined to be 25 participants. Considering potential participant lost follow up or withdrawal, we set a loss to follow up rate of 20 %. Based on this assumption, we estimated a total of 31 participants was required.

### Experimental protocol

This study consisted of 3 phases: preparation, CPR under the experimental condition, and outcome measurement after the experimental condition. This study involved two experimental sessions. First, chest compressions were performed under normal conditions with WBGT 21℃ and then one week later, chest compressions were performed under hot and humid conditions with WBGT 31℃.

(1) Preparation

Prior to the study, the baseline characteristics of the participants (height, weight, BMI, heart rate, blood pressure, core body temperature, and blood lactate levels) were recorded. To accurately and continuously measure core body temperature, participants ingested a radiotelemetry pill (e-Celsius® Performance, BodyCap, France) four hours before the experiment began and refrained from eating or drinking during that period. This radiotelemetry pill can measure and monitor body temperature continuously in real-time via Bluetooth. It ensures high safety and has been utilized for research and sports applications to measure a core body temperature^15^, ^16^.

(2) CPR under the designated condition

Before the experiment, we checked that the health status of the participants on the day of experiment and they had had no symptoms or conditions related to COVID-19. In this study, we used the Wet Bulb Globe Temperature (WBGT) as an index to represent hot and humid environments. WBGT is calculated by combining three elements: Wet Bulb Temperature, Globe Temperature, and Dry Bulb Temperature, and it is commonly used to assess the risk of heat-related illnesses. A WBGT of 31 or higher indicates “Exercise is essentially prohibited,” 28–31 is “Extreme caution,” 25–28 is “Be alert,” 21–25 is “Caution,” and 21 or lower is “Mostly safe.” The higher the WBGT, the greater the risk of heat stress^17^. In this study, 1based on data from the Japanese Ministry of the Environment's “Relationship between Heat Index and heat-related illness Transport Cases in 2020,” we calculated the average WBGT on days with over 1,000 transport cases and designated WBGT 31 as the hot and humid environment^18^. The control group was set at WBGT 21. For Tokyo, we defined the “normal” environment as 20.5 °C with 50.5 % humidity (WBGT 21 °C), and the “hot and humid” environment as 37.0 °C with 64.0 % humidity (WBGT 31 °C).

**Fig. 1:**
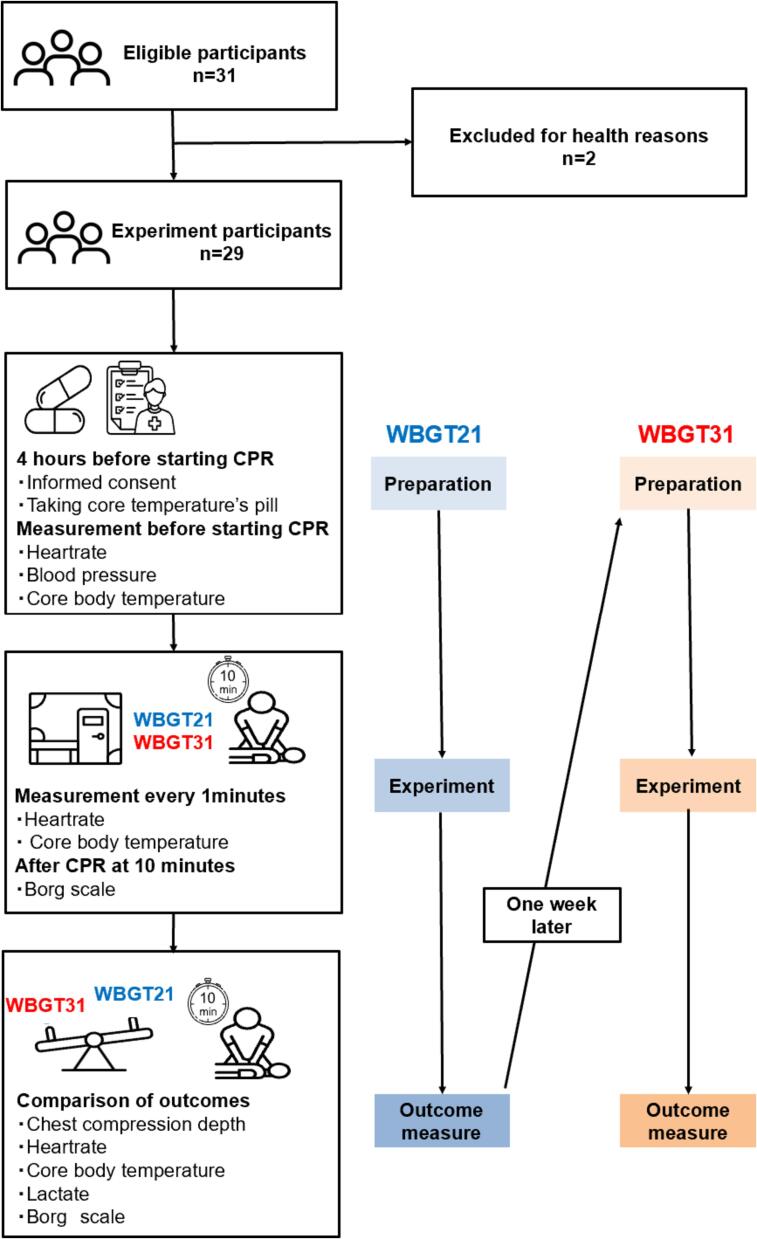
Study flowchart. Schema of an experiment to determine whether physical load and fatigue levels are induced by performing chest compressions in a high-temperature environment. Each participant experienced different locations, WBGT21℃. and WBGT31℃. WBGT21 is normal condition with WBGT21℃, WBGT31 is hot and humid condition with WBGT31℃. WBGT: Wet-Bulb Globe Temperature. CPR: Cardiopulmonary resuscitation.

Subsequently, each participant performed hands-only CPR alone for 10 min in the artificial climate chamber. A simulator mannequin (Laerdal Medical, Stavanger, Norway) was used for the chest compressions. During the chest compressions, heart rate and core body temperature were measured every minute. At the end of the experiment, participants were asked to rate their perceived fatigue using the Borg scale, which is a commonly used scale to assess the intensity of exercise and physical load by themselves. The scale ranges from 6 to 20, where 6 indicates “very light” exercise and 20 indicates “very hard” exercise. Blood lactate levels were measured before and after the study using the blood sampled from the participants' fingertips (Blood Lactate Meter Lactate Pro 2 LT-173, Arkray, Kyoto, Japan). An overview of the experiment is shown in Fig. 1.

(3) Criteria for termination

The criteria for termination of this experiment were based on either subjective complaints from the participants and physiological parameters. Termination criteria based on subjective complaints included the presentation of heat illness symptoms, chest pain, or a desire by the subject to stop. The physiological criteria for termination included a core body temperature reaching 40 °C. The study was conducted under the supervision of a paramedic (certified life-saving technician), and if the paramedic deemed it necessary to stop, the experiment was also terminated. For the safety management of participants and heat-related illness prevention, after the experiment, the vital signs of the participants were measured in the same manner as before the experiment, and heat-related illness prevention measures were implemented.

(4) Outcome measurement

The primary outcome was the depth of chest compressions over a 10-minute period measured by the mannequin mentioned above. The secondary outcomes were the changes in heart rate, core body temperature during CPR and, perceived fatigue using the Borg scale, and blood lactate levels after the 10 min of chest compressions.

### Statistical analysis

We used Two-way Repeated Measures Analysis of Variance (ANOVA) to assess whether differences in WBGT affect the depth of chest compressions, heart rate, and core body temperature. The Borg scale and blood lactate levels differences before and after the experiment using paired t-tests. We have refrain from using p-value for the secondary outcomes, indicating the mean difference and confidence interval instead. The statistical analysis was performed using R software (version 4.0.2, The R Foundation for Statistical Computing, Vienna, Austria). A p-value of less than 0.05 was considered statistically significant.

## Results

### Participant background

Out of 31 participants, two were excluded due to their health conditions on the day of the experiment, leaving 29 participants for analysis. The participants' mean age [SD] was 21 years [0.7], consisting of 21 males (70.5 %) and 8 females (29.5 %). The participants' height, weight, BMI, and CPR training history are shown in Table 1. The study flowchart is indicated in Fig. 1.

**Table 1:** Characteristics.

	Overall (n = 29)
Age, (years)	21 (0.7)
Male, (%)	21 (70.5)
Height, (cm)	168.0 (7.3)
Weight, (kg)	63.0 (7.6)
BMI, (kg/m^2^)	22.3 (1.9)
CPR training history, (time)	3 (1.9)

For continuous variables, use mean and standard deviation (SD); for categorical variables, use percentage (%) and case count.

Age, gender, grade, height, weight, and BMI were the study subject characteristic items.

IQR: Interquartile Range, SD: Standard Deviation.

BMI: Body Mass Index.

CPR: Cardiopulmonary Resuscitation.

### Chest compressions

**Fig. 2:**
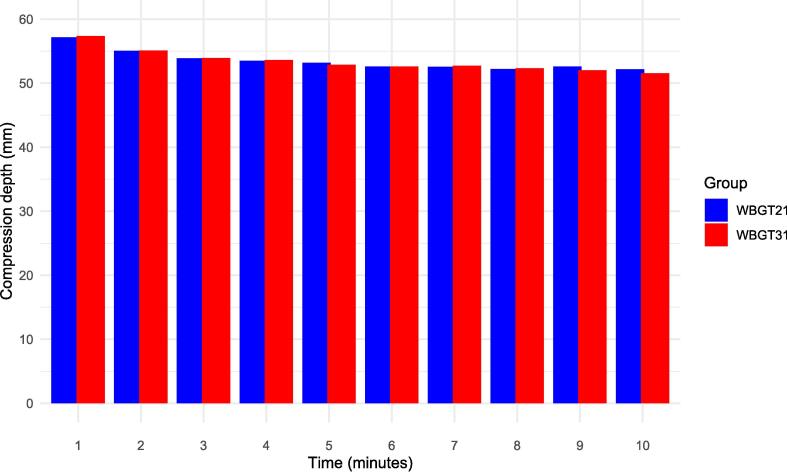
Comparison chest compression depths. This figure compares the chest compression during 10 min at WBGT 21℃ and WBGT 31℃. The mean [SD] depth of chest compression were as follows, WBGT 21℃: 57.2 mm [5.1] vs or WBGT 31℃: 57.4 mm [4.0] at the 1 min, WBGT 21℃: 53.2 mm [7.1] vs or WBGT 31℃: 52.9 mm [6.8] at the 5 min, and WBGT 21℃: 52.2 mm [7.4] vs or WBGT 31℃: 51.5 mm [7.6] at the 10 min. There was no significant difference in chest compression depth between the WBGT 21℃ and WBGT 31℃ groups (p = 0.52). WBGT: Wet-Bulb Globe Temperature. WBGT21 is normal condition with WBGT21℃, WBGT31 is hot and humid condition with WBGT31℃. WBGT: Wet-Bulb Globe Temperature.

The depth of chest compressions is shown in Fig. 2. The mean [SD] depth of chest compression were as follows, WBGT 21℃: 52.2 mm [7.4] vs or WBGT 31℃: 51.5 mm [7.6] at the 10 min. There was no significant difference in chest compression depth between the WBGT 21℃ and WBGT 31℃ groups (p = 0.52).

### Heart rate and core body temperature

**Fig. 3:**
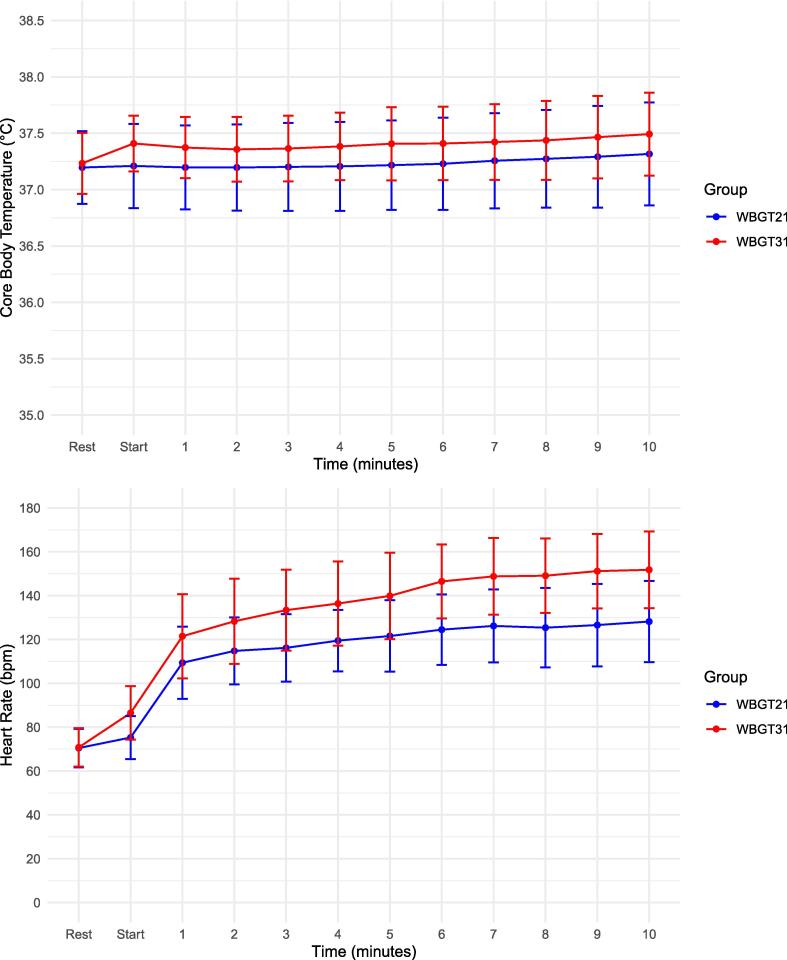
Comparison heartrate and core body temperature WBGT21℃ vs WBGT31℃. This figure compares the heart rates and core body temperature during 10 min of chest compressions at WBGT 21℃ and WBGT 31℃. WBGT21 is normal condition with WBGT21℃, WBGT31 is hot and humid condition with WBGT31℃. HR: Heart rate, bpm: beats per minute. WBGT: Wet-Bulb Globe Temperature.

Heart rate and core body temperature are shown in Fig. 3. At 10 min, heart rate between WBGT 21℃ and WBGT 31℃ was 126 bpm vs 143 bpm (mean difference [95 % CI ]: 17 bpm [7.7–26.3]).

Additionally, At 10 min, core body temperature between WBGT 21℃ and WBGT 31℃ was 37.4℃ vs 37.5℃ (mean difference [95 % CI]: 0.1℃ [-0.1–0.3]).

### Borg scale and blood lactate levels

The Borg scale is shown in Table 2. At the end of the experiment, Borg scale between WBGT 21℃ and WBGT 31℃ was 16 “very hard” vs 18 “extremely hard” (mean difference [95 % CI]: 2 [1–3]).

**Table 2:** Borg scale at the end of experiment.

	WBGT21	WBGT31	p-value
Borg scale at the end of experiment	16 (2)	18 (2)	<0.001

This figure shows the Borg scale ratings after 10 min of chest compressions at WBGT 21℃ and WBGT 31℃.

For Borg scale use mean and standard deviation (SD).

WBGT21 is normal condition with WBGT21℃, WBGT31 is hot and humid condition with WBGT31℃.

WBGT: Wet-Bulb Globe Temperature.

SD: Standard Deviation.

**Fig. 4:**
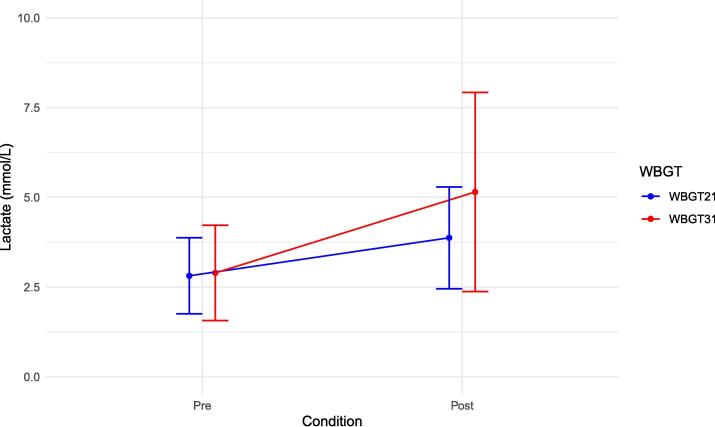
Comparison of lactate before and at the end of chest compressions. This figure compares the lactate before and after 10 min of chest compressions at WBGT 21℃ and WBGT 31℃. WBGT21 is normal condition with WBGT21℃, WBGT31 is hot and humid condition with WBGT31℃. WBGT: Wet-Bulb Globe Temperature.

The blood lactate levels is shown in Fig. 4. At the end of the experiment, blood lactate levels between WBGT 21℃ and WBGT 31℃ was 3.9 vs 5.1 (mean difference [95 % CI]: 1.2 mmol/L [0.1–2.3]).

## Discussion

### Key observations

No significant differences were observed in the depth of chest compressions between environments with WBGT21℃ and WBGT31℃; however, participants under the WBGT31℃ had increased heart rate, core body temperature, Borg scale scores, and blood lactate levels, compared to WBGT21℃. This study has demonstrated that the environment significantly increased the physiological load of bystanders during CPR, which has not previously been well investigated.

### Interpretation

There are several potential explanations for this result. We hypothesized that CPR in hot and humid environments may lead to a decline in the quality of chest compressions; however, in this study, we found no difference in the quality of chest compressions between WBGT 21℃ and WBGT 31℃. We speculated that this result may be because the participants in this study were healthy volunteers and paramedic university students who had received substantial CPR training. Previous studies which reported that individuals who have received CPR training can maintain a certain level of chest compression quality^19^. While no difference in chest compression quality was observed in this study, the results might vary depending on the participants, and further investigation is needed.

Second, this study revealed that bystander CPR in hot and humid environments imposes significant physiological stress on rescuers. In this study, the blood lactate level after chest compressions in a WBGT 31℃ environment was 5.1 [2.8] mmol/L. The increase in blood lactate levels in hot and humid environments is believed to result from physiological mechanisms and heightened exercise intensity under these conditions. In such environments, body temperature rises, prompting the body to dissipate heat, which leads to increased breathing and heart rate to meet the body's oxygen demands^20^, ^21^. However, as blood flow shifts to the skin to cool the body, blood flow to the muscles decreases, resulting in insufficient oxygen supply for the muscles. This oxygen deficit forces the muscles to rely on anaerobic metabolism, promoting lactate production, which accumulates and increases fatigue. These physiological processes likely contribute to elevated blood lactate levels during CPR in hot and humid environments. Additionally, regarding exercise intensity under different environmental conditions, we assumed that it differed between WBGT 21 and WBGT 31 conditions. Heart rate is commonly used to measure exercise intensity, and as intensity increases, heart rate rises to meet the muscles' oxygen demands^22^. In this study, heart rate was significantly higher under WBGT 31 conditions, indicating that exercise intensity was higher during CPR in WBGT 31. This increase in exercise intensity may have contributed to the rise in blood lactate levels. These findings suggest that bystander CPR in hot and humid environments imposes significant physiological stress on rescuers.

Third, chest compressions in a hot and humid environment resulted in increased cardiovascular impact and fatigue. In the previous study by Bridgewater et al., participants (mean age of 44 years) from an emergency service department with CPR training experience had a peak heart rate of 128 bpm during 10 min of chest compressions^5^. The result in their study showed the similar to that observed in our study (126 bpm in the WBGT 21 °C environment) among CPR-experienced individuals. In contrast, in the hot and humid environment (WBGT 31 °C), the peak heart rate rose to 143 bpm, indicating that the cardiac and physical load on CPR providers significantly increased under these conditions. Additionally, previous studies have reported that in hot and humid environments, as body temperature rises, blood flow to the skin increases while blood flow to the muscles decreases, leading to insufficient oxygen supply to the muscles^20^, ^21^. As a result, muscles switch to anaerobic metabolism, promoting lactate production and accumulation, which increases fatigue. In this study, we observed increases in lactate levels and Borg scale ratings in the WBGT 31 environment. Our findings on increased fatigue in hot and humid environments are likely consistent with these previous studies, suggesting that chest compressions in hot and humid environments significantly increase perceived fatigue for rescuers.

### Clinical implication

We consider that this study has two main clinical implications. First, in this study, no significant difference in chest compression depth was observed between WBGT 31℃ and WBGT 21℃ conditions among young paramedic students volunteers who had received sufficient CPR training. This result contradicts the initial hypothesis; however, it indicates that trained rescuers could maintain a consistent quality of CPR for 10 min, even in hot and humid environments. This finding suggests that appropriate CPR could be delivered by trained paramedic to cardiac arrest patients in regions with hot and humid summer, which may be crucial evidence to consider the quality of CPR in the settings where the climate is changing. In contrast, this result generate the further research question whether general people without routine CPR training could perform chest compressions with the same quality even in hot and humid environments. Our study could be used as a justification for the further study on the CPR under hot and humid environment.

Additionally, as a secondary outcome, it was found that heart rate, blood lactate levels, and fatigue scores based on the Borg scale increased under the WGBT31 conditions. Although these are results from the exploratory analysis as the secondary outcomes, it would suggest that performing CPR in a hot and humid environment keeping chest compression depth could result in high physical exertion, leading to physiological stress, which may affect the safety and performance of rescuers in such environments. Based on these results, we would suggest that the interval for switching rescuers during CPR in hot and humid environments. Furthermore, it is important to ensure proper hydration and rest breaks to reduce the risk of heat related illness for rescuers. Although further research should be warranted, this result would suggest the novel research direction to the health impact of CPR providers under the hot and humid environment. We believe these points should be relevant as the climate change is happening globally.

### Limitation

This study has several limitations. First, the use of a simulator manikin for the experiments may differ from OHCA patients in clinical settings. Although simulator manikins are widely used in chest compression research, they do not replicate the characteristics of the thorax, such as stiffness and resistance, found in actual human bodies. This difference may influence the effort required for CPR. Second, this study involved only young, healthy volunteers who were paramedic university students receiving training to become paramedics. Therefore, the data might differ from general citizens, where bystanders might be middle-aged or older. Future research should focus on the load and fatigue experienced during chest compressions across different age groups. Third, this study used a simple blood lactate meter to measure blood lactate levels from the participants' fingertips, which may be less accurate compared to blood lactate values obtained through conventional arterial blood sampling. Fourth, this study was conducted during the summer, so it is possible that the participants had acclimatized to the hot and humid environment. Although current evidence on heat acclimatization is insufficient, it is believed that the results may have been influenced by the individual levels of acclimatization. Fifth, this study was conducted in a climate chamber, and it presents different environmental conditions compared to actual emergency scenes. In real situations, bystanders may be exposed to various external factors such as wind, noise, temperature and surrounding environments. Accordingly, it is important to be careful in interpreting the results of this study and applying the results to clinical settings, and further research is necessary. Finally, the multiple outcomes in this study may pose a risk of multiplicity. The primary outcome is the p-value for chest compression depth, while the other p-values are positioned as exploratory outcomes. For exploratory outcomes, it is important to recognize the risk of multiplicity.

## Conclusion

In this study, there was no significant difference in the depth of chest compression of paramedic students under the conditions between WBGT 31℃ and WBGT 21℃. For secondary outcomes, the lactate and fatigue of bystanders increased under WBGT 31℃ compared to WBGT 21℃. Further research is needed on CPR in hot and humid environments.

## Founding

This study was supported by Japan Foundation for Emergency Medicine and the ZOLL Foundation.

## CRediT authorship contribution statement

**Haruka Takahashi:** Writing – original draft, Visualization, Methodology, Investigation, Funding acquisition, Formal analysis, Data curation, Conceptualization. **Kensuke Suzuki:** Writing – review & editing, Project administration, Methodology, Investigation, Formal analysis, Data curation, Conceptualization. **Yohei Okada:** Writing – review & editing, Supervision, Formal analysis. **Satoshi Harada:** Writing – review & editing, Supervision, Methodology. **Hiroyuki Yokota:** Writing – review & editing, Supervision. **Marcus Eng Hock Ong:** Writing – review & editing, Supervision. **Satoo Ogawa:** Writing – review & editing, Supervision.

## Declaration of competing interest

The authors declare the following financial interests/personal relationships which may be considered as potential competing interests: [Haruka Takahashi has received a research grant from Japan Foundation for Emergency Medicine and ZOLL Foundation. Yohei Okada has received a research grant from the ZOLL Foundation and an overseas scholarship from the FUKUDA foundation for medical technology and the International medical research foundation. Hiroyuki Yokota is an advisor to Aioi Nissay Dowa Insurance Co., Ltd. This organization has no role in conducting this research. Marcus Eng Hock Ong reports grants from the Laerdal Foundation, Laerdal Medical, and Ramsey Social Justice Foundation for funding of the Pan-Asian Resuscitation Outcomes Study an advisory relationship with Global Healthcare SG, a commercial entity that manufactures cooling devices; and funding from Laerdal Medical on an observation program to their Community CPR Training Centre Research Program in Norway. Marcus Eng Hock Ong is a Scientific Advisor to TIIM Healthcare SG and Global Healthcare SG. Marcus Eng Hock Ong is a member of the editorial board of Resuscitation. These organizations have no role in conducting this research. All other authors reported no competing interests].
